# Taxonomic novelties in *Helminthosphaeriaceae*: morphological and phylogenetic evidence for *Obovatispora* gen. nov. and a new species from *Polygonatum* in Guizhou Province, China

**DOI:** 10.3897/mycokeys.132.182249

**Published:** 2026-05-26

**Authors:** Xiao-Fang Chen, Xiang-Yu Zhang, Qing-Song Yuan, Jian Ma, Tao Zhou

**Affiliations:** 1 Resource Institute for Chinese & Ethnic Materia Medica, Guizhou University of Traditional Chinese Medicine, Guiyang, Guizhou 550025, China Bijie Medical College Bijie China; 2 Bijie Medical College, Bijie, Guizhou 551700, China Resource Institute for Chinese & Ethnic Materia Medica, Guizhou University of Traditional Chinese Medicine Guiyang China; 3 Bijie Institute of Traditional Chinese Medicine, Bijie, Guizhou 551700, China Bijie Institute of Traditional Chinese Medicine Bijie China; 4 Guizhou Key Laboratory for Germplasm Innovation and Resource-Efficient Utilization of Dao-di Herbs, Guiyang, Guizhou 550025, China Guizhou Key Laboratory for Germplasm Innovation and Resource-Efficient Utilization of Dao-di Herbs Guiyang China; 5 Guizhou Industry Polytechnic College, Guiyang, Guizhou 550008, China Guizhou Industry Polytechnic College Guiyang China

**Keywords:** Asexual morph, phylogeny, *
Polygonatum
*, *
Sordariomycetes
*, taxonomy

## Abstract

*Obovatispora* is established herein as a new genus to accommodate a novel hyphomycetous species, *O. polygonati*, collected from dead branches of *Polygonatum* in Guizhou Province, China. Phylogenetic analyses based on a concatenated LSU, ITS, and *tef*1-α dataset place *Obovatispora* within *Helminthosphaeriaceae* (*Chaetosphaeriales*, *Sordariomycetidae*). The genus is presently known only from its asexual morph and is characterized by macronematous, unbranched, septate, cylindrical conidiophores; mono- or polyblastic, cylindrical conidiogenous cells that occasionally bear lateral, bladder-like conidiogenous loci; and acropleurogenous, pyriform, obovoid to broadly ovoid conidia. This study provides detailed morphological descriptions, illustrations, and comparative assessments of the new taxon. The discovery of *O. polygonati* not only enriches the taxonomic framework of *Helminthosphaeriaceae* but also enhances understanding of species diversity and biogeographical associations linked to the medicinal plant genus *Polygonatum*.

## Introduction

Guizhou Province, located in southwestern China, features highly heterogeneous topography, varied microclimatic conditions, and well-preserved natural ecosystems, which collectively contribute to its status as a major biodiversity hotspot (Yang et al. 2023; Zhang et al. 2025). In recent years, intensified field investigations and systematic sampling by mycologists and ecologists have led to the continual discovery of previously undocumented fungal taxa from this region (Lu et al. 2018; Yang et al. 2023; Liu et al. 2024; Ma et al. 2024; Lin et al. 2025; Sun et al. 2025; Zhang et al. 2025). These discoveries encompass a wide range of ecological and trophic groups, including aquatic and terrestrial fungi, endophytes inhabiting healthy host tissues, plant pathogens, and saprobes colonizing decaying substrates (Lu et al. 2018; Yang et al. 2023; Liu et al. 2024; Ma et al. 2024; Lin et al. 2025; Zhang et al. 2025; Xiao et al. 2025). Among these research efforts, considerable attention has been devoted to the taxonomy and systematics of freshwater fungi, resulting in the description of a substantial number of new species and genera from submerged woody substrates (Hyde et al. 2016, 2020, 2021; Su et al. 2016; Bao et al. 2018, 2019, 2020, 2021, 2023; Luo et al. 2018, 2019; Dong et al. 2020, 2021a, 2021b; Calabon et al. 2021, 2022, 2023; Shen et al. 2022a, 2022b, 2024; Xu et al. 2023, 2024, 2025; Yang et al. 2023; Wang et al. 2024a, 2024b, 2025; Xiao et al. 2025). In addition to freshwater taxa, several new species have also been isolated from medicinal plants, demonstrating the unique flora of Guizhou as an important reservoir for uncovering fungal diversity (Sun et al. 2025; Zhang et al. 2025). Collectively, this expanding body of taxonomic and phylogenetic research underscores the pivotal role of Guizhou Province as a key region for fungal discovery, systematics, and biodiversity conservation.

*Chaetosphaeriales (Sordariomycetidae)* was introduced based on LSU sequence data (Huhndorf et al. 2004). Currently, this order comprises six families, viz., *Chaetosphaeriaceae*, *Helminthosphaeriaceae*, *Leptosporellaceae*, *Linocarpaceae*, *Neoleptosporellaceae*, and *Pseudocapsulosporaceae* (Hongsanan et al. 2017; Konta et al. 2017; Wijayawardene et al. 2020; Hyde et al. 2024). The family *Helminthosphaeriaceae (Chaetosphaeriales)* was established by Samuels et al. (1997), with *Helminthosphaeria* designated as the type genus. Later, Hyde et al. (2024) provided a comprehensive outline of fungi and fungus-like organisms, in which *Helminthosphaeriaceae* was circumscribed to include six genera: *Echinosphaeria*, *Helminthosphaeria*, *Hilberina*, *Kramasamuha*, *Ruzenia*, and *Selenosporella*. Previous studies have suggested the inclusion of additional genera in this family; however, these were not incorporated into the 2024 outline (Miller et al. 2014; Hernández-Restrepo et al. 2017; Chuaseeharonnachai et al. 2023; Hyde et al. 2024). For example, *Endophragmiella* and *Synaptospora* have been placed in *Helminthosphaeriaceae* based on consistent morphological characteristics and supporting molecular phylogenetic evidence (Cain 1957; Miller et al. 2014; Hernández-Restrepo et al. 2017; Chuaseeharonnachai et al. 2023). With these additions, the family is currently considered to comprise eight genera (Cain 1957; MacGarvie 1968; Subramanian and Vittal 1973; Hilber and Hilber 2002; Miller et al. 2014; Bell and Mahoney 2016; Hernández-Restrepo et al. 2017; Chuaseeharonnachai et al. 2023; Bispo et al. 2024). Among these genera, *Endophragmiella* is notable for its highly variable conidial morphology, producing conidia that range from clavate and fusiform to ellipsoidal, or even Y-shaped (Hernández-Restrepo et al. 2017; Chuaseeharonnachai et al. 2023).

During a survey of fungi associated with medicinal plants in Guizhou Province, China, a previously undescribed taxon was discovered. Phylogenetic analyses based on a combined dataset of LSU, ITS, and *tef*1-α sequences revealed that the newly collected specimens form a well-supported, distinct lineage within the family *Helminthosphaeriaceae*. By integrating detailed morphological examinations with multigene phylogenetic evidence, *Obovatispora* is proposed as a new genus to accommodate the unique taxon *O. polygonati*. A comprehensive taxonomic treatment is provided, including detailed morphological descriptions, high-resolution illustrations, and comparative analyses with morphologically and phylogenetically related genera. This discovery not only enriches the known taxonomic diversity of saprobic fungi associated with medicinal plants in Guizhou but also contributes to a deeper understanding of the ecological relationships and biogeographical patterns linking saprobic fungal assemblages to their medicinal plant hosts in karst landscapes.

## Materials and methods

### Sample collection, isolation, and morphological studies

Fresh specimens were collected from a terrestrial habitat in Yinjiang Tujia and Miao Autonomous County, Tongren City, Guizhou Province, China, on 23 March 2025. The specimens were stored in Ziplock bags, labeled with a marker pen, and examined under a stereomicroscope (SMZ 745, Nikon, Japan). The collection, examination, and isolation protocols followed those established by Senanayake et al. (2020) and Rathnayaka et al. (2024). Micromorphological characters were captured using a Nikon EOS 90D digital camera attached to an ECLIPSE Ni compound microscope (Nikon, Japan). Morphological measurements were carried out utilizing the Tarosoft (R) Image Frame Work tool (version IFW 0.97), while photoplates were constructed employing Adobe Photoshop 2019 software (Adobe Systems, USA).

Following the morphological examination, the specimens were archived at the herbaria of the Guizhou Academy of Agriculture Sciences (GZAAS), Guiyang, China. Additionally, ex-type living cultures were preserved at the Guizhou Culture Collection (GZCC). Species recognition and the justification for establishing the new species were based on the guidelines outlined by Chethana et al. (2021).

### DNA extraction, PCR amplification, and sequencing

Mycelium freshly scraped from living cultures was transferred to 1.5 mL microcentrifuge tubes and stored in a refrigerator at –20 °C. Genomic DNA extraction was carried out using DNA extraction kits provided by Sangon Biotech (Shanghai) Co. Ltd., China. Polymerase chain reaction (PCR) was employed for DNA template amplification, utilizing established primer pairs: LR0R/LR5 for LSU (Vilgalys and Hester 1990; Cubeta et al. 1991), ITS5/ITS4 for ITS (White et al. 1990), and EF1-983F/EF1-2218R for *tef*1-α (Rehner and Buckley 2005). PCR was conducted in a 50 μL reaction volume, comprising DNA template (2 μL), forward primer (2 μL), reverse primer (2 μL), 2 × Taq PCR Master Mix (25 μL), and 19 μL of double-distilled water. The thermal-cycling parameters for the LSU, ITS, and *tef*1-α regions were as follows: initial denaturation at 94 °C for 3 min, followed by 40 cycles of denaturation at 94 °C for 45 s, annealing at 56 °C for 50 s, elongation at 72 °C for 1 min, and final extension at 72 °C for 10 min. PCR product verification was performed on 1% agarose gels before submission to Sangon Biotech (Shanghai) Co., Ltd., China, for sequencing.

### Phylogenetic analyses

The forward and reverse sequences from the newly generated sequences were assembled using the Contig Express v3.0.0 application. Reference sequences used in this study (Table 1) were downloaded from the NCBI GenBank database (https://blast.ncbi.nlm.nih.gov/Blast.cgi). Each individual dataset was aligned using the online version of MAFFT v. 7 (https://mafft.cbrc.jp/alignment/server/index.html) (Katoh et al. 2017). The LSU, ITS, and *tef*1-α alignments were trimmed using trimAl v1.2rev59 (Capella-Gutiérrez et al. 2009) and subsequently concatenated using SequenceMatrix v1.7.8 (Vaidya et al. 2011).

**Table 1.** Taxa used in this study and their GenBank accession numbers for LSU, ITS, and *tef*1-α sequence data.

**Taxon**	**Strain**	**GenBank accessions**		
		**LSU**	**ITS**	***tef*1-α**
*Ascomycota* sp.	GZAAS 20-4004	OP099545	N/A	OR140429
*Chaetosphaeria decastyla*	CBS 142446	OR134630	OR134686	OR130766
***Chloridium aquaticum***	**MFLUCC 11-0212**	**MH476567**	**MH476570**	**N/A**
***Chloridium aseptatum***	**MFLUCC 11-0216**	**MH476568**	**NR_158365**	**N/A**
***Claviformispora phyllostachydis***	**SICAUCC 16-0004**	**MT232720**	**MT232736**	**MT240855**
*Cryptophiale hamulata*	MFLUCC 18-0098	MG386756	N/A	N/A
*Cryptophiale udagawae*	MFLUCC 18-0422	MH758211	MH758198	N/A
*Cryptophiale udagawae*	MFLUCC 18-0428	MH758210	MH758197	N/A
*Dictyochaeta assamica*	CBS 242.66	MH870426	MH858788	N/A
***Dictyochaeta pandanicola***	**MFLUCC 17-0563**	**MH376710**	**MH388338**	**MH388373**
***Dictyochaeta siamensis***	**MFLUCC 15-0614**	**KX609952**	**KX609955**	**N/A**
***Dictyochaeta terminalis***	**GZCC 18-0085**	**MN104624**	**MN104613**	**N/A**
***Echinosphaeria canescens***	**SMH4666**	**KF765605**	**N/A**	**N/A**
*Echinosphaeria canescens*	SMH4791	AY436403	N/A	N/A
*Endophragmiella dimorphospora*	FMR 12150	KY853502	KY853442	N/A
***Escovopsis multiformis***	**LESF 850**	**OQ589785**	**OQ589835**	**OQ603928**
*Gelasinospora tetrasperma*	CBS 178.33	DQ470980	NR_077163	DQ471103
***Helminthosphaeria clavariarum***	**SMH4609**	**AY346283**	**N/A**	**N/A**
***Helminthosphaeria leptospora***	**ILLS 00121778**	**NG_243230**	**N/A**	**N/A**
*Helminthosphaeriaceae* sp.	BRA CR40346	PV052810	N/A	N/A
***Hilberina caudata***	**SMH1542**	**KF765615**	**N/A**	**N/A**
***Infundibulomyces cupulata***	**BCC11929**	**EF113979**	**EF113976**	**N/A**
***Infundibulomyces oblongisporus***	**BCC13400**	**EF113980**	**EF113977**	**N/A**
***Kionochaeta castaneae***	**GZCC 18-0025**	**MN104621**	**MN104610**	**N/A**
***Kionochaeta microspora***	**GZCC 18-0036**	**MN104618**	**MN104607**	**N/A**
*Kramasamuha sibika*	COAD:2632	MN794358	MN794381	N/A
*Kramasamuha sibika*	CBS 146133	MN794357	MN794380	N/A
*Kramasamuha* sp.	XG276a	PV578356	PV578192	N/A
***Leptosporella arengae***	**MFLUCC 15-0330**	**MG272246**	**MG272255**	**MG272259**
***Leptosporella bambusae***	**MFLUCC 12-0846**	**KU863122**	**KU940134**	**N/A**
***Leptosporella cocois***	**MFLUCC 15-0816**	**N/A**	**MG272256**	**N/A**
***Leptosporella elaeidis***	**MFLU 19-0669**	**MK659772**	**MK659767**	**MN883560**
***Leptosporella gregaria***	**SMH4290**	**AY346290**	**N/A**	**N/A**
*Leptosporella gregaria*	SMH4673	HM171287	N/A	N/A
***Linocarpon arengae***	**MFLUCC 15-0331**	**MG272247**	**N/A**	**N/A**
***Linocarpon cocois***	**MFLUCC 15-0812**	**MG272248**	**MG272257**	**N/A**
*Menispora tortuosa*	DAOM 231154	AY544682	KT225527	N/A
*Menispora tortuosa*	CBS 214.56	AF178558	AF178558	N/A
***Menisporopsis anisospora***	**CBS 109475**	**MH874421**	**MH862827**	**N/A**
***Menisporopsis breviseta***	**GZCC 18-0071**	**MN104623**	**MN104612**	**N/A**
***Menisporopsis dushanensis***	**GZCC 18-0084**	**MN104626**	**MN104615**	**N/A**
***Menisporopsis pandanicola***	**KUMCC 17-0271**	**MH376726**	**MH388353**	**MH388388**
*Menisporopsis theobromae*	MFLUCC 15-0055	KX609954	KX609957	N/A
***Neoleptosporella palmae***	**HKAS 115694**	**NG_245577**	**NR_200698**	**N/A**
***Neolinocarpon arengae***	**MFLUCC 15-0323**	**MG272249**	**MG272258**	**N/A**
*Neolinocarpon phayaoense*	MFLUCC 17-0073a	MG581933	N/A	MG739512
*Neolinocarpon phayaoense*	MFLUCC 17-0073b	MG581934	N/A	MG739513
***Neolinocarpon phayaoense***	**MFLUCC 17-0074**	**MG581935**	**N/A**	**MG739514**
***Neolinocarpon rachidis***	**MFLUCC 15-0332**	**MG272250**	**N/A**	**N/A**
*Neolinocarpon rachidis*	MFLUCC 15-0814a	MK106353	MK106342	N/A
*Neolinocarpon rachidis*	MFLUCC 15-0814b	MK106354	N/A	N/A
***Obovatispora polygonati***	**GZCC 25-27588**	**PX848713**	**N/A**	**PZ095455**
*Obovatispora polygonati*	GZCC 25-27589	PX848714	N/A	PZ095456
***Phialosporostilbe scutiformis***	**MFLUCC 17-0227**	**MH758207**	**MH758194**	**N/A**
*Phialosporostilbe scutiformis*	MFLUCC 18-1288	MH758212	MH758199	N/A
***Pseudocapsulospora phoenicis***	**MFLU 24-0170**	**NG_245589**	**NR_200712**	**N/A**
***Pseudocapsulospora rhapidis***	**GZCC 21-0242**	**NG_245590**	**NR_200713**	**N/A**
***Ruzenia spermoides***	**SMH4606**	**AY436422**	**N/A**	**N/A**
*Ruzenia spermoides*	SMH4655	KF765619	N/A	N/A
*Selenosporella curvispora*	CBS 102623	MW144405	N/A	N/A
*Selenosporella falcata*	CBS 659.75	MH872731	MH860962	N/A
*Sordaria fimicola*	CBS 508.50	MH868251	MH856730	N/A
*Synaptospora plumbea*	ANM963	KF765620	N/A	N/A
*Synaptospora plumbea*	SMH3962	KF765621	N/A	N/A
***Zanclospora iberica***	**FMR 11584**	**KY853544**	**KY853480**	**N/A**
*Zanclospora iberica*	FMR 12186	KY853545	KY853481	N/A

Note: All sequences from ex-type strains are in bold. The newly generated sequence is in red bold. “N/A” indicates data unavailable in GenBank.

Phylogenetic analyses in this study were conducted using RAxML-HPC v.8 on XSEDE (8.2.12) under the GTRGAMMA model with rapid bootstrap analysis and 1,000 bootstrap replicates (Stamatakis 2014). The best-fit substitution model was automatically tested by the server. Bayesian inference (BI) was performed using the “MrBayes on XSEDE” tool (Huelsenbeck and Ronquist 2001; Swofford 2002; Stamatakis et al. 2008; Miller et al. 2010; Ronquist et al. 2012). These analyses were based on a combined dataset of LSU and ITS sequences.

Model selection for each gene region in the Bayesian analysis was performed using MrModeltest v2. The aligned FASTA file was converted to Nexus format using AliView v. 1.27 (Daniel et al. 2010) for subsequent Bayesian analysis. Phylogenetic trees were visualized using FigTree version 1.4.0 and further edited using Adobe Photoshop 2019 program (Adobe Systems, USA) and Adobe Illustrator version 51.1052.0.0 (Adobe Inc., San Jose, California, USA).

### Phylogenetic analysis results

The multilocus phylogenetic analyses (LSU, ITS, and *tef*1-α) were conducted to elucidate the phylogenetic position of the newly obtained strains. The dataset, consisting of DNA sequences from 66 taxa with a total alignment length of 2,397 characters (including gaps: LSU: 1–883 bp, ITS: 884–1,458 bp, and *tef*1-α: 1,459–2,397 bp), was used for the phylogenetic analyses. These analyses were conducted using maximum likelihood (ML) and BI, with *Gelasinospora tetrasperma* (CBS 178.33) and *Sordaria fimicola* (CBS 508.50) as outgroups. ML and BI analyses of the concatenated LSU, ITS, and *tef*1-α datasets yielded similar tree topologies. The best-fit substitution model selected for the BI analysis was GTR+I+G for the LSU and *tef*1-α partitions, whereas GTR+G was applied to the ITS partition. Base frequencies and rates were A = 0.226759, C = 0.281348, G = 0.308527, and T = 0.183365; substitution rates were AC = 1.082963, AG = 1.730846, AT = 1.032984, CG = 0.996716, CT = 5.140888, and GT = 1.000000. The gamma distribution shape parameter (*α*) was 0.321458.

**Figure 1.**
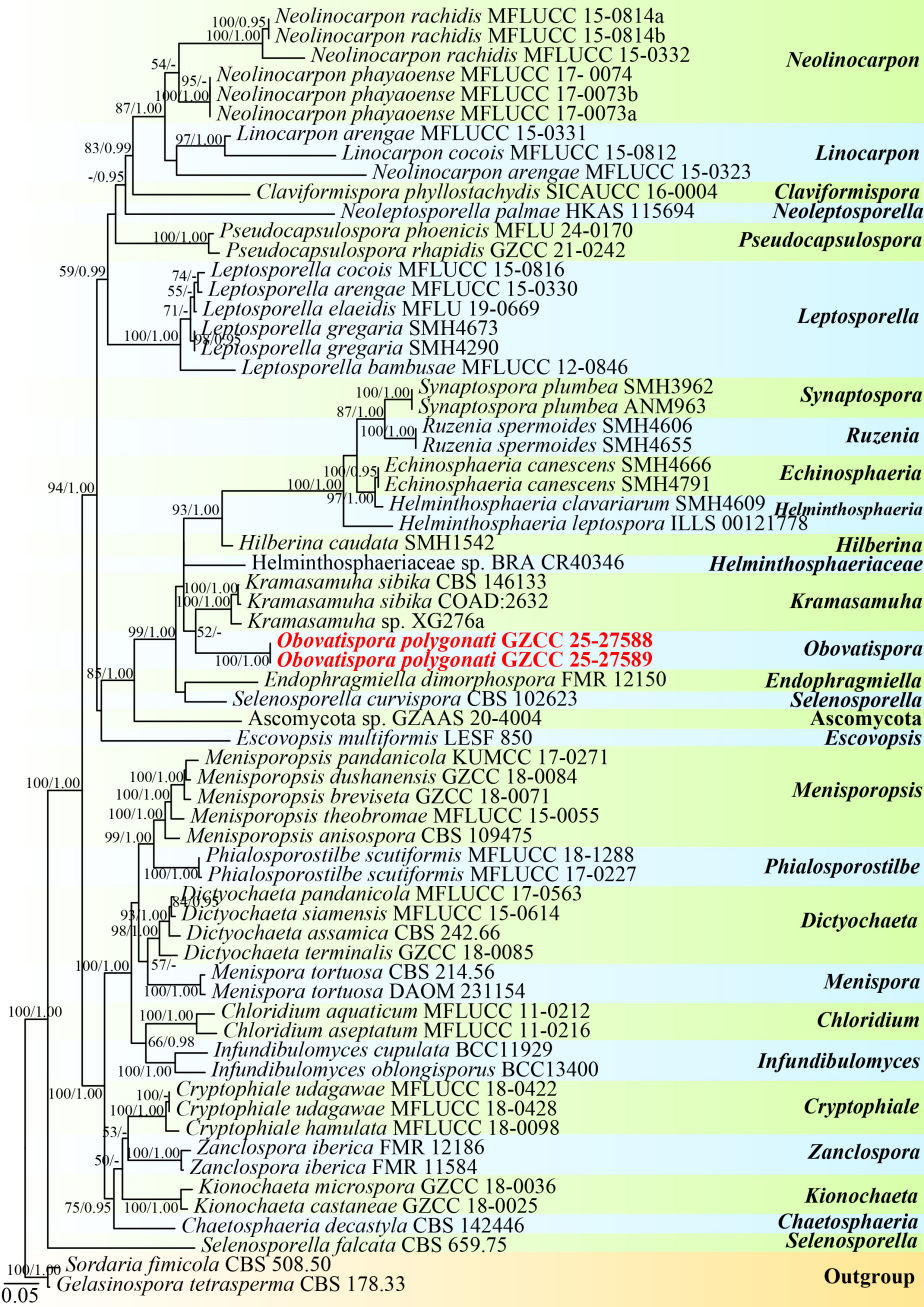
Phylogenetic analysis of *Obovatispora* was conducted using RAxML-based maximum likelihood analyses of a combined LSU, ITS, and *tef*1-α DNA sequence dataset. Bootstrap support values for ML equal to or greater than 60% (ML left) and Bayesian posterior probabilities (BI right) equal to or greater than 0.95 are shown above the nodes. A hyphen (“-”) indicates a value lower than 60% for ML and a posterior probability lower than 0.95 for Bayesian. The tree is rooted with *Gelasinospora tetrasperma* (CBS 178.33) and *Sordaria fimicola* (CBS 508.50). Newly generated strains are highlighted in red bold.

Based on the multigene phylogenetic tree (Fig. 1), the collections represent a new genus and a novel species within *Helminthosphaeriaceae* (*Chaetosphaeriales*, *Sordariomycetidae*). The two isolates (GZCC 25-27588 and GZCC 25-27589) form a sister clade to the lineage comprising *Kramasamuha sibika* (CBS 146133 and COAD:2632) and *Kramasamuha* sp. (XG276a), with an unstable bootstrap value.

## Taxonomy

### Obovatispora

Taxon classificationFungiAsparagalesAsparagaceae

X.F. Chen, X.Y. Zhang & T. Zhou gen. nov.

4E706FBD-619D-5108-91E9-54325442C13B

904727

#### Etymology.

“*Obovatispora*” refers to the obovoid-broadly ovoid conidia characteristic of this genus.

#### Description.

***Saprobic*** on dead branches of ***Polygonatum***. ***Asexual morph***: ***Colonies*** superficial, effuse, hairy, velvety, brown to black, scattered. ***Mycelium*** partly superficial, partly immersed, consisting of branched, septate, smooth, smooth-walled, hyaline to pale brown hyphae. ***Conidiophores*** macronematous, mononematous, solitary, erect, unbranched, septate, straight or flexuous, cylindrical, slightly constricted at septa, brown, becoming subhyaline to pale brown towards the apex. ***Conidiogenous cells*** mono- or polyblastic, integrated, terminal or intercalary, occasionally bearing bladder-like conidiogenous loci at the subapical cylindrical, subhyaline to brown. ***Conidia*** acropleurogenous, pyriform, obovoid to broadly ovoid, 1-septate, guttulate, subhyaline to brown, slightly constricted at the septa, with a subhyaline remain of the conidiogenous cell and small basal protrusion at the base of the conidia, smooth-walled. ***Sexual morph***: Undetermined.

#### Type species.

*Obovatispora polygonati* X.F. Chen, X.Y. Zhang & T. Zhou

#### Notes.

Phylogenetically, *Obovatispora* forms a distinct clade within *Helminthosphaeriaceae* (*Chaetosphaeriales*, *Sordariomycetidae*), supporting its recognition as a new genus. Morphologically, *Obovatispora* is readily distinguished from other genera in the family by its unbranched conidiophores, conidiogenous loci that are bladder-like or cylindrical, and 1-septate, pyriform, obovoid to broadly ovoid conidia. Here, *Obovatispora* is established to accommodate a new species, *O. polygonati*, which is designated as the type species based on both molecular evidence and its distinctive conidiophores, conidiogenous cells, and conidial characteristics.

### Obovatispora polygonati

Taxon classificationFungiAsparagalesAsparagaceae

X.F. Chen, X.Y. Zhang & T. Zhou sp. nov.

73FB2F5A-81D6-5043-87EE-3212F62E7E21

904728

Fig. 2

#### Etymology.

“polygonati’’ refers to the medicinal plant *Polygonatum*, from which the fungus was collected.

**Figure 2.**
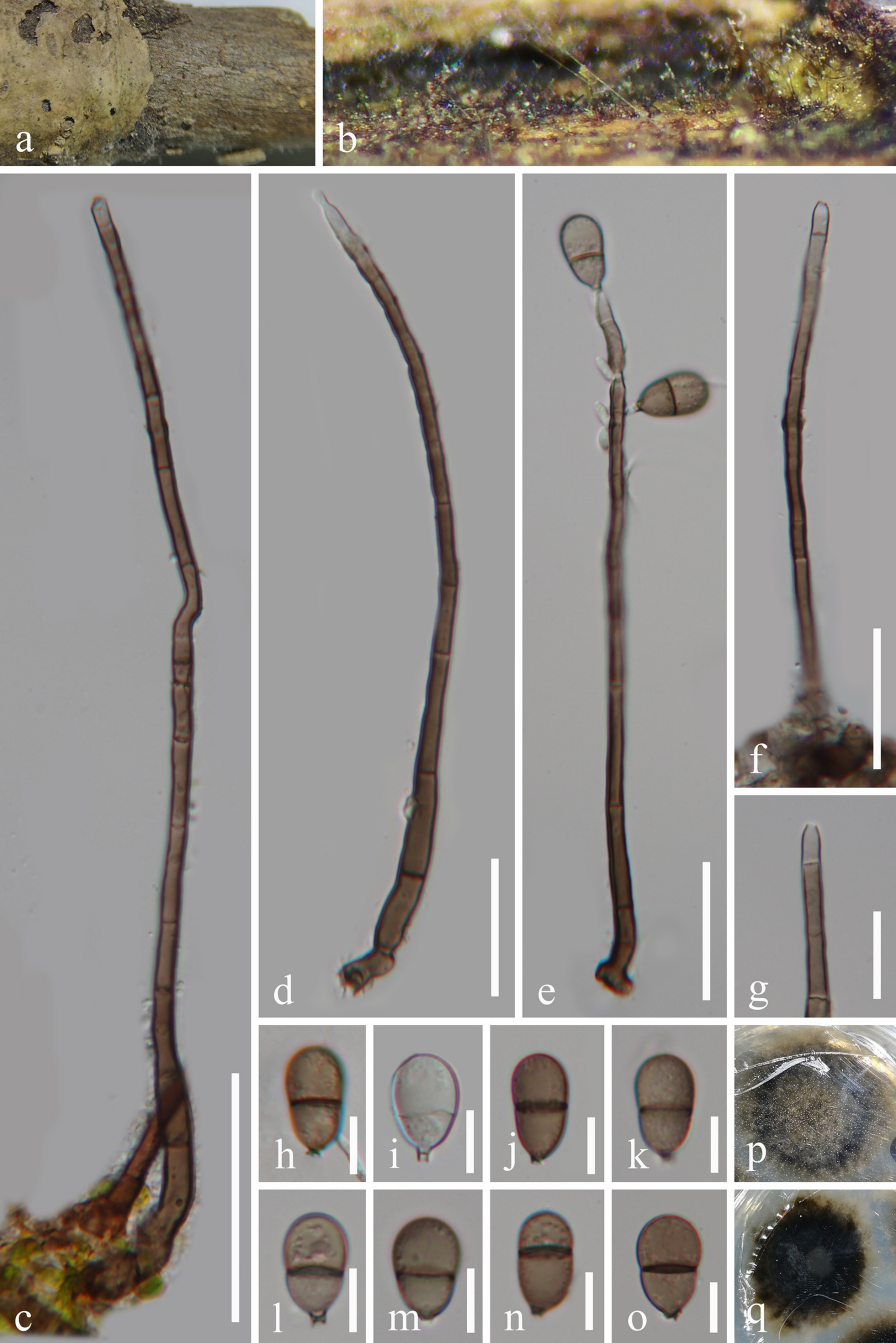
*Obovatispora polygonati* (GZAAS 25-0779, holotype). **a, b**. Colonies on the host surface; **c, d, f**. conidiophores and conidiogenous cells; **e**. conidiophores, conidiogenous cells, and conidia; **g**. conidiogenous cell; **h**. germinated conidium; **i–o**. conidia; **p, q**. colonies on PDA from above and below after 34 days of incubation at room temperature. Scale bars: 50 μm (**c**); 30 μm (**d–f**); 20 μm (**g**); 10 μm (**h–o**).

#### Holotype.

GZAAS 25-0779

#### Description.

***Saprobic*** on dead branches of ***Polygonatum***. ***Asexual morph***: ***Colonies*** superficial, effuse, hairy, velvety, brown to black, scattered. ***Mycelium*** partly superficial, partly immersed, consisting of branched, septate, smooth, smooth-walled, hyaline to pale brown hyphae. ***Conidiophores*** 105–230 × 5–7.3 μm (x̄ = 160.7 × 6 μm, ***n*** = 25), macronematous, mononematous, solitary, erect, unbranched, septate, straight or flexuous, cylindrical, slightly constricted at septa, brown, becoming subhyaline to pale brown towards the apex. ***Conidiogenous cells*** 7.2–13 × 2.7–4.2 μm (x̄ = 9.5 × 3.4 μm, ***n*** = 30), mono- or polyblastic, integrated, terminal or intercalary, occasionally bearing bladder-like conidiogenous loci at the subapical, 5–5.5 × 2.1–2.4 μm, cylindrical, subhyaline to brown. ***Conidia*** 15.4–20.4 × 9.2–12.2 μm (x̄ = 17.7× 10 μm, ***n*** = 30), acropleurogenous, pyriform, obovoid to broadly ovoid, 1-septate, guttulate, subhyaline to brown, slightly constricted at the septa, with a subhyaline remain of the conidiogenous cell and small basal protrusion at the base of the conidia, smooth-walled. ***Sexual morph***: Undetermined.

#### Culture characteristics.

Conidia germinated on PDA within 13 h, producing germ tubes from the conidial body. Colonies on PDA were irregular with a flat surface and an undulate margin, reaching 31 mm in diameter after 34 days at room temperature (approximately 25 °C). Colony coloration was pale brown to black on both the surface and reverse sides.

#### Material examined.

China • Guizhou Province, Tongren City, Yinjiang Tujia and Miao Autonomous County, on dead branches of *Polygonatum*, 23 March 2025, Xiao-Fang Chen, Xiang-Yu Zhang & Tao Zhou, HJ10 (GZAAS 25-0779, holotype), ex-type living culture GZCC 25-27588; • ibid., HJ6 (GZAAS 25-0780, paratype), living culture GZCC 25-27589.

#### Notes.

In the phylogenetic tree (Fig. 1), the isolates (GZCC 25-27588 and GZCC 25-27589) formed a distinct sister clade to *Kramasamuha sibika* (CBS 146133 and COAD:2632) and *Kramasamuha* sp. (XG276a), although this relationship is supported by unstable bootstrap support. Comparative analyses of LSU sequences revealed that the collection (GZCC 25-27588) differs from *K. sibika* (CBS 146133) by 58 out of 827 bp (7%, including three gaps). In addition, GZCC 25-27588 differs from *Kramasamuha* sp. (XG276a) by 56 out of 871 bp (6.4%, including three gaps). Morphologically, *Obovatispora polygonati* (GZAAS 25-0660) can be readily distinguished from *Kramasamuha sibika* by its conidiogenous cells, which are single and bladder-like to cylindrical, whereas those of *K. sibika* are mostly arranged in clusters of 2–4 and are lageniform to almost cylindrical (Subramanian and Vittal 1973). *Obovatispora polygonati* possesses predominantly cylindrical conidiogenous cells, sometimes with tiny bladder-like conidiogenous loci, whereas those of *K. sibika* are ampulliform and curved or taper nearly to a point at the apex (Subramanian and Vittal 1973; Bispo et al. 2024). Therefore, based on multigene phylogenetic evidence combined with its distinct morphological characteristics, *O. polygonati* is designated as a novel species within the genus *Obovatispora*.

## Discussion

The newly introduced genus *Obovatispora* shares morphological features with *Endophragmiella* and *Helminthosphaeria*, particularly in the structure of conidiophores, conidiogenous cells, and conidia (Cain 1957; MacGarvie 1968; Subramanian and Vittal 1973; Hilber and Hilber 2002; Miller et al. 2014; Bell and Mahoney 2016; Hernández-Restrepo et al. 2017; Chuaseeharonnachai et al. 2023; Bispo et al. 2024). However, *Obovatispora* can be readily distinguished by its uniquely shaped obovoid to broadly ovoid conidia, which contrast with the predominantly cylindrical to ellipsoidal conidia of *Endophragmiella* and the more variable conidial morphologies observed in *Helminthosphaeria* (Samuels et al. 1997; Hernández-Restrepo et al. 2017; Chuaseeharonnachai et al. 2023). This distinctive conidial morphology, together with molecular phylogenetic evidence, supports the recognition of *Obovatispora* as a separate genus within *Helminthosphaeriaceae*. The combination of morphological uniqueness and phylogenetic distinctness underscores the importance of integrating classical taxonomy with molecular systematics for resolving evolutionary relationships within the family.

Medicinal plants play a crucial role in the prevention and treatment of human diseases, largely due to their rich repertoire of bioactive compounds with diverse pharmacological properties, including antimicrobial, antioxidant, and anti-inflammatory activities (Schmidt 2017; Rahman et al. 2019). These plants also serve as hosts to complex microbial communities, encompassing endophytic, pathogenic, and saprobic fungi, which contribute to plant health, nutrient cycling, and overall ecosystem functioning (Atanasov et al. 2021). To date, research has predominantly focused on endophytic fungi because of their potential as sources of novel bioactive metabolites and their intimate associations with living plant tissues (Zhang et al. 2025). In contrast, pathogenic and saprobic fungi associated with medicinal plants have received comparatively little attention, despite their critical ecological roles in decomposition, nutrient recycling, and the structuring of plant–fungal interactions (Du et al. 2024). Expanding investigations to include these fungal groups is therefore essential for a more comprehensive understanding of the diversity, ecological significance, and evolutionary relationships of plant-associated fungi.

Previous phylogenetic studies have demonstrated that the taxonomic placement of *Helminthosphaeria* species within *Helminthosphaeriaceae* remains problematic and far from resolved (Crous et al. 2023). Several species traditionally assigned to *Helminthosphaeria* do not cluster together in molecular analyses. For example, *Helminthosphaeria carpathica*, *Synaptospora plumbea*, and *Ruzenia spermoides* consistently form a distinct sister clade; *H. stuppea* groups with *Echinosphaeria canescens*; and *Endophragmiella dimorphospora*, *H. hispidissima*, and *H. tomaculum* cluster together as a separate lineage (Crous et al. 2023). These phylogenetic patterns indicate that *Helminthosphaeria* is not monophyletic and may comprise multiple evolutionarily distinct lineages. The results further support the notion that *Helminthosphaeria* is taxonomically unstable and potentially polyphyletic, revealing marked discrepancies between morphology-based classifications and molecular phylogenetic evidence. Such inconsistencies highlight the need for expanded taxon sampling, the incorporation of additional molecular markers, and a comprehensive morphological re-evaluation to resolve the systematics of this complex genus.

## Supplementary Material

XML Treatment for Obovatispora

XML Treatment for Obovatispora polygonati

## Additional information

### Artificial Intelligence (AI) use

The authors accept full responsibility for the content of the manuscript, including the disclosure of any use of AI.

No AI tools were used in the preparation of this manuscript.

### Author ORCIDs

X.-F. Chen https://orcid.org/0009-0000-9962-7644

X.-Y. Zhang https://orcid.org/0009-0009-7330-9651

Q.-S. Yuan https://orcid.org/0000-0002-9713-099X

J. Ma https://orcid.org/0009-0008-1291-640X

T. Zhou https://orcid.org/0000-0001-8508-9355

### Data availability

All of the data that support the findings of this study are available in the main text.
